# Gaze transition entropy as a measure of attention allocation in a dynamic workspace involving automation

**DOI:** 10.1038/s41598-024-74244-4

**Published:** 2024-10-08

**Authors:** Zixin Cui, Tetsuya Sato, Austin Jackson, Sampath Jayarathna, Makoto Itoh, Yusuke Yamani

**Affiliations:** 1 University of Tsukuba, Tsukuba, Japan; 2 Old Dominion University, Norfolk, USA

**Keywords:** Computer science, Information technology, Psychology, Human behaviour

## Abstract

Real-world work environments require operators to perform multiple tasks with continual support from an automated system. Eye movement is often used as a surrogate measure of operator attention, yet conventional summary measures such as percent dwell time do not capture dynamic transitions of attention in complex visual workspace. This study analyzed eye movement data collected in a controlled a MATB-II task environment using gaze transition entropy analysis. In the study, human subjects performed a compensatory tracking task, a system monitoring task, and a communication task concurrently. The results indicate that both gaze transition entropy and stationary gaze entropy, measures of randomness in eye movements, decrease when the compensatory tracking task required more continuous monitoring. The findings imply that gaze transition entropy reflects attention allocation of operators performing dynamic operational tasks consistently.

## Introduction

Modern professional environments require visual multi-tasking to support complex human-machine interaction in aviation^1^, driving^2,3^, healthcare^4^, military^5^, and nuclear plant operation^6^. For example, an operator of unmanned aerial vehicle is required to monitor the state of the aircraft while tracking the trajectory of other aircrafts in airspace^7^. In such operational environment, operators are required to perform multiple tasks in which operators are supported by automated technology, ever increasing complexity and dynamism of the work environment and requiring successful human-automation interaction. The literature indicates that humans can be conceptualized as resource-limited information processors^8^, imposing a tradeoff between task demand and performance limits. Multiple concurrent tasks thus complete for the limited attentional resources influencing different information-processing stages including sensory processing, perception, working/long-term memory, decision making, and action selection/implementation^9^, resulting in performance losses in human operators.

Previous research supports that eye movements can serve as a proxy to allocation of attentional resources in supervisory control tasks^2,10^. For example, experimental and computational modeling works show that the probability that an operator fixates a particular area of interest (AOI) varies as a function of factors that are known to drive attention such as saliency^11^ and expectancy and value^12–14^ (see Wickens et al.^15^ for a fuller description of the SEEV model). The primary measure of visual attention in these studies is percent dwell time (PDT) which refers to the sum of the time that the gaze coordinates fall within an AOI, providing a global numerosity measure of eye movement^16^.

Recent works characterized operator’s attention allocation strategies when performing multiple tasks^17,18^. For example, Sato et al.^18^ examined whether interruption frequency impacted attention allocation in attention-demanding environment. Participants were asked to perform a MATB-II synthetic task comprising of a compensatory tracking task, a communication task, and a system monitoring task that was assisted by a 70%-reliable automated aid. Task load was manipulated by the difficulty of the tracking task while interruption frequency was manipulated by the number of auditory commands in the communication task (4 vs. 16 interruptions). Results indicated that participants attended less toward the system monitoring task under high multitasking demand. Additionally, participants attended the communication task more frequently when the communication task interrupted more frequently. These results indicated that task load and task interruption can account for a shift of attention. A caveat when interpreting these data, though, is that the previous studies mainly used PDT as a measure of attentional resources^2,17–19^, which only captures global gaze distribution without considering gaze shift between AOIs. One potential way to capture the nuanced nature of attention allocation in multitasking environment is to measure gaze transition entropy.

### Gaze transition entropy analysis

Gaze transition entropy is a measure of randomness in gaze transitions between AOIs indicating gaze dispersion and visual exploration within a given task. Gaze transition entropy has been found to have diverse applications across domains such as surgical procedures^20^, flight control^21^, and driving tasks^22^. For example, in aviation, decreased gaze entropy during solving in-flight emergencies reflects a more focused and consistent visual scanning among pilots^21^. Gaze entropy can be measured by SGE and GTE. SGE measures the uncertainty of fixation locations within a given viewing time, reflecting the overall spatial dispersion of gaze that results from a given level of scanning efficiency. GTE estimates uncertainty not only in total spatial dispersion but also in the sequential pattern of visual scanning capturing the dynamic allocation of attentional resources. GTE was designed to statistically compare fixation transitions between AOIs. Since SGE and GTE assess distinct aspects of visual scanning, concurrently evaluating both could offer a more comprehensive evaluation of gaze behavior and attention allocation^23,24^. Beyond more conventional eye movement measures, such as percent dwell time and areas of interest (AOIs), gaze entropy measures can quantify the spatial and temporal randomness of visual scanning. That is, the gaze entropy measures capture more information about the complexity and dynamic nature of gaze dispersion across the visual field than the other available measures, better suited for applied tasks that require scanning across multiple AOIs for a prolonged period of time. Following previous studies^25,26^, SGE and GTE can be respectively computed using Eqs. (1) and (2).

1 $$\begin{aligned} H_s(x)= & -\sum _{i}^{n} P_i\log _2 P_i \end{aligned}$$

2 $$\begin{aligned} H_t(x)= & -\sum _{i}^{n} P_i \sum _{j}^{n} P_{ij}\log _2P_{ij} \end{aligned}$$

where $p_i$ is the observed (simple) probability of viewing the $i{\textrm{th}}$ AOI, $p_{ij}$ is the conditional probability of transitioning from the $i{\textrm{th}}$ to the $j{\textrm{th}}$ AOI. $H_t$ indicates the predictability of gaze transitions; a high $H_t$ ($\forall _{ij}|p_{ij}{\rightarrow }{0.5}$) implies low predictability, whereas a low $H_t$ ($\forall _{ij}|p_{ij}{\rightarrow }\{0,1\}$) implies high predictability. See ^27^ for a good discussion on the Gaze Transtition Entropy and details of the calculations.

Previous work examined gaze entropy in an attention-demanding environment^28^. Ayala et al.^28^ examined the effect of task difficulty on gaze entropy in a simulated flight environment whereby the landing task involved a strong wind (i.e., difficult condition) or a weak wind (i.e., easy condition). Results showed that participants exhibited low GTE and SGE scores under the difficult condition, indicating that participants’ gaze behavior became less random and more predictable when the task demanded more sensorimotor control. Yet, none of the prior work examined whether task interruption influences gaze entropy in an attention-demanding environment.

### Current study

The present paper applies *gaze transition entropy analysis* to an operator’s eye data to provide additional insights into the allocation of attentional resources in multitasking environments. Specifically, the present study reanalyzed Sato et al.’s^18^ eye movement data to explore how gaze transition entropy (GTE) and stationary gaze entropy (SGE) manifest in the attention-demanding environment. We hypothesized that participants would exhibit lower GTE and SGE scores when the central tracking task demanded more attention^28^ or when the frequency of the auditory interruption was higher.

## Results

Data of one participant were excluded from Sato et al.’s^18^ study because the error rate in the system monitoring task was above the threshold (i.e., 50%).

### Gaze entropy

**Fig. 1:**
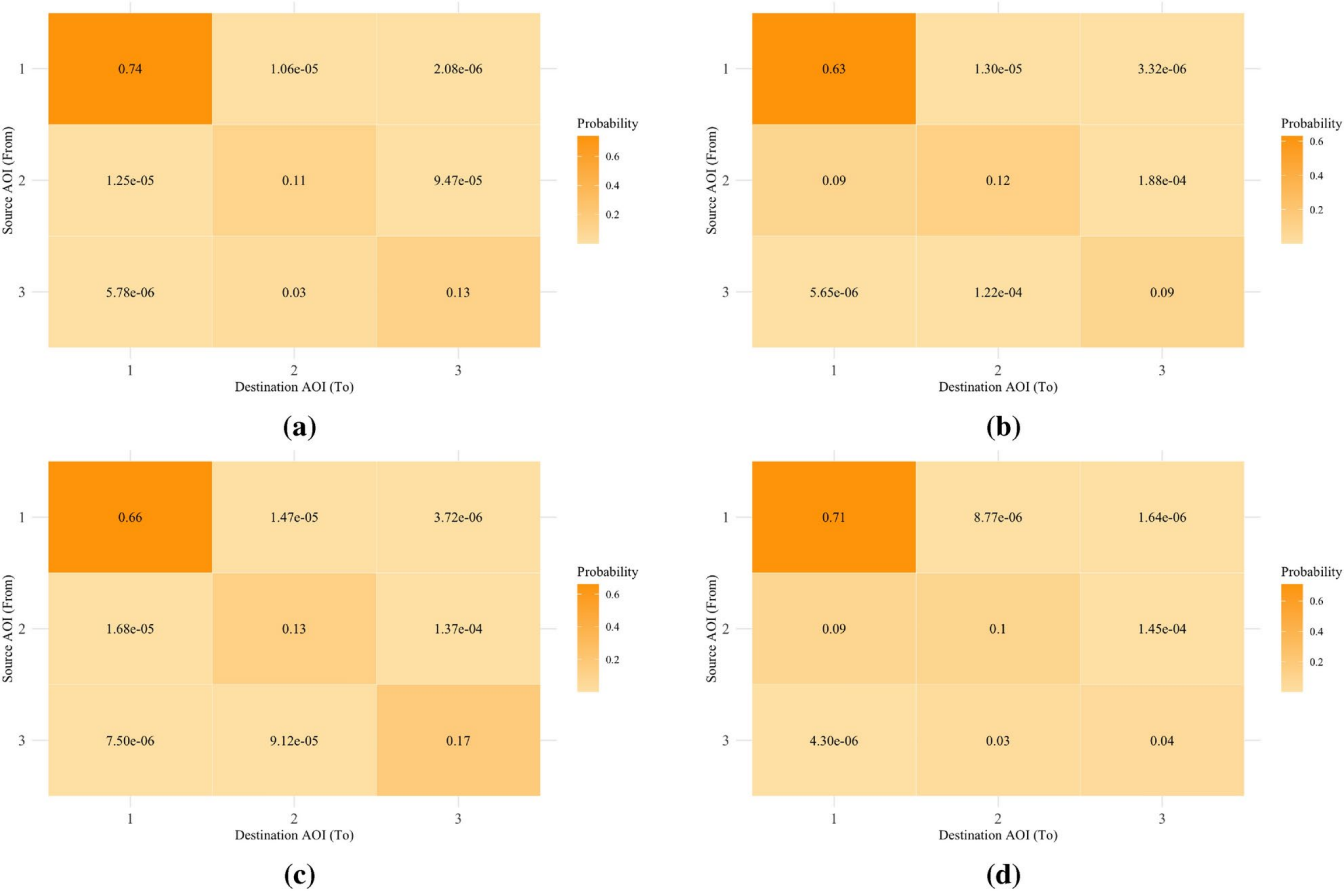
Gaze transition probability matrices reflecting the transition of each source AOI (1 = tracking task, 2 = system monitoring task, 3 = communication task) to destination AOI (1= tracking task, 2 = system monitoring task, 3 = communication task). Values in matrices (**a**) and (**b**) indicate gaze transition probabilities in Difficult and Easy conditions, respectively. Values in matrices (**c**) and (**d**) indicate gaze transition probabilities in the High and Low Communication Load conditions, respectively.

**Fig. 2:**
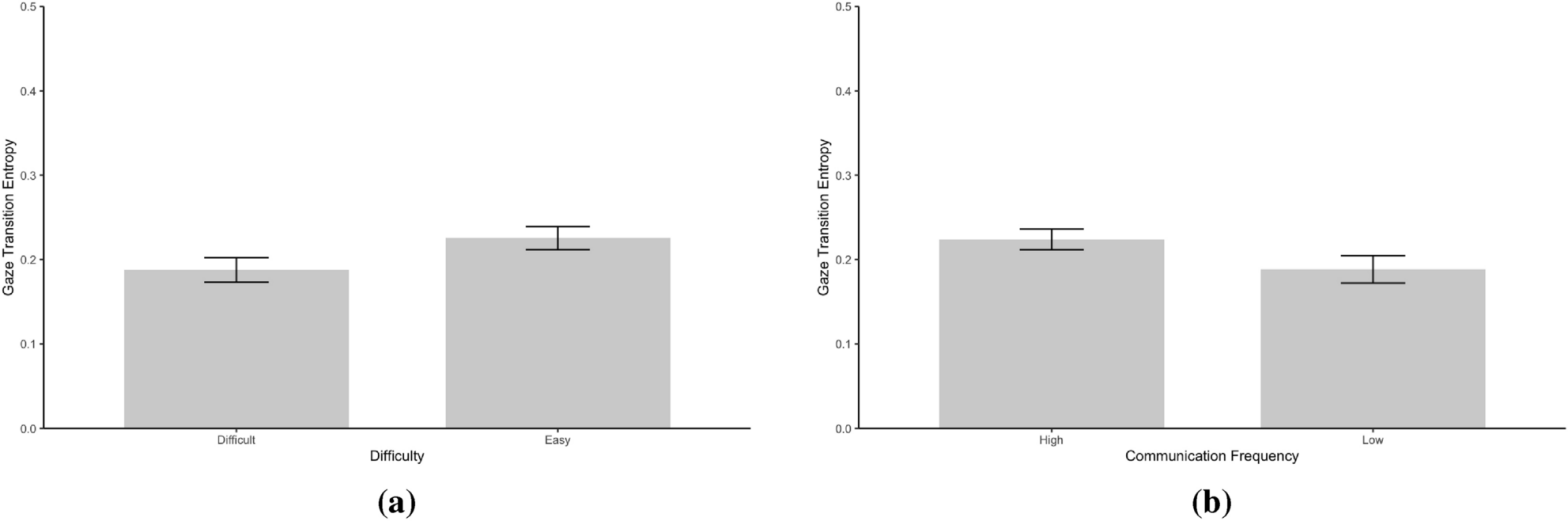
Gaze transition entropy between Difficulty (**a**) and Communication Frequency (**b**) conditions. Error bars represent 95% confidence intervals.

**Fig. 3:**
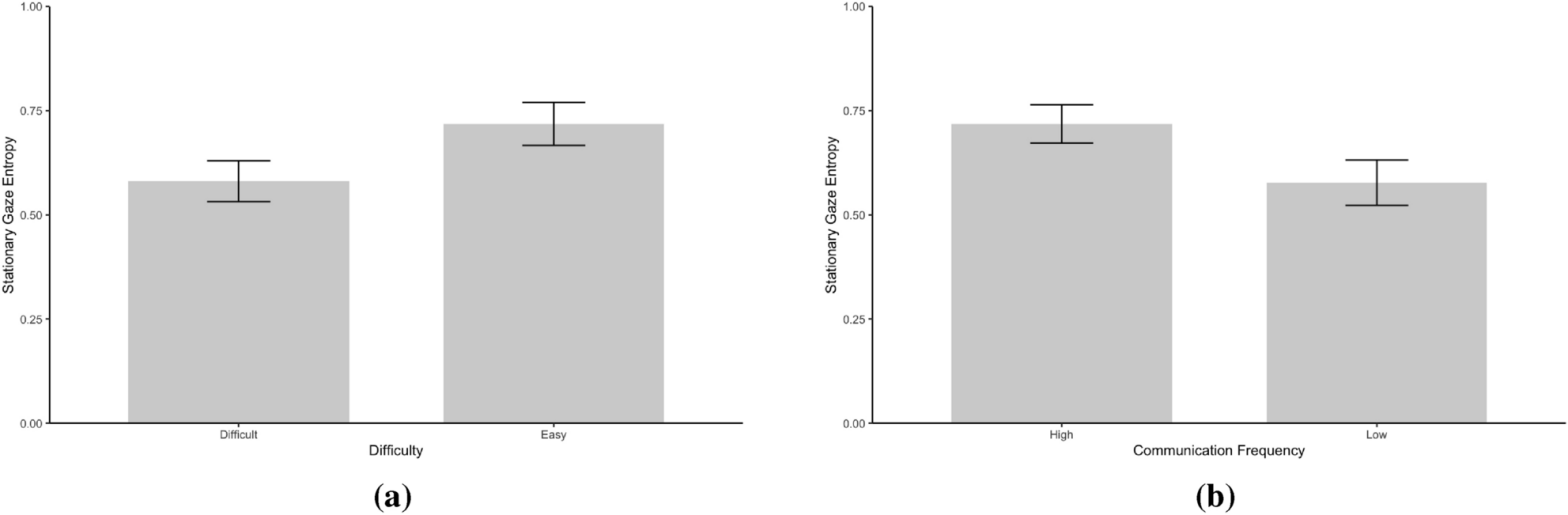
Stationary gaze entropy between Difficulty (**a**) and Communication Frequency (**b**) conditions. Error bars represent 95% confidence intervals.

The present study analyzed gaze transition entropy and stationary gaze entropy. Figure 1 presents gaze transition probability matrices between Difficulty conditions and between Communication Frequency conditions. Figure 2 presents GTE between Difficulty and Communication Frequency conditions. Figure 3 presents SGE between Difficulty and Communication Frequency conditions.

#### Gaze transition probability

As shown in Fig. 1a, b, data presented decisive evidence that participants were more likely to transition their gaze away from the tracking task under the Easy condition than the Difficult condition [*F*(1, 37) = 67.72, $BF_{10}$ = $1.79 \times 10^7$, $\eta ^2_G$ = 0.22]. Also, there was decisive evidence that participants were more likely to transition their gaze from the system monitoring task to the communication task under the Easy condition than the Difficult condition [*F*(1, 37) = 20.82, $BF_{10}$ = 665.12, $\eta ^2_G$ = 0.13]. However, the likelihood that participants transitioned their gaze away from the system monitoring task did not substantially differ between Difficulty conditions [*F*(1, 37) = 0.04, $BF_{10}$ = 1/4.13, $\eta ^2_G$ < 0.01]. Additionally, gaze transition probability from the communication task to the tracking task did not substantially differ between Easy condition and Difficult condition [*F*(1, 37) = 0.01, $BF_{10}$ = 1/4.39, $\eta ^2_G$ < 0.01]. In Fig. 1c, d, there was substantial evidence that participants were more likely to transition their gaze away from the communication task under the High communication frequency condition than the Low communication frequency condition [*F*(1, 37) = 9.13, $BF_{10}$ = 5.96, $\eta ^2_G$ = 0.12]. For the remaining cell values, there were no evidence on whether gaze transition probabilities differed between conditions of Difficulty and Communication Frequency [1/2.63 < $BF_{10}$ < 1.13]. Furthermore, there was no evidence for and against the interaction effect on all transition patterns [1/2.93 < $BF_{10}$ < 1/1.56].

#### Gaze transition entropy

Data gave decisive evidence that participants exhibited greater GTE under the Easy condition than the Difficult condition [*M* = 0.23 vs. 0.19; *F*(1, 37) = 53.63, $BF_{10}$ = $9.88 \times 10^5$, $\eta ^2_G$ = 0.19]. Additionally, data indicate substantial evidence that GTE was higher in the High communication frequency condition compared to the Low communication frequency condition [*M* = 0.22 vs. 0.19; *F*(1, 37) = 9.08, $BF_{10}$ = 8.64, $\eta ^2_G$ = 0.17]. However, results showed no substantial evidence for the two-way interaction effect between Difficulty and Communication Frequency on GTE [*F*(1, 37) = 3.36, $BF_{10}$ = 1.04, $\eta ^2_G$ = 0.01].

#### Stationary gaze entropy

Data indicate decisive evidence that participants exhibited greater SGE under the Easy condition than the Difficult condition [*M* = 0.72 vs. 0.58; *F*(1, 37) = 73.94, $BF_{10}$ = $2.79 \times 10^7$, $\eta ^2_G$ = 0.21]. Data showed very strong evidence that participants exhibited higher SGE in the High communication frequency condition than those in the Low communication frequency condition [*M* = 0.72 vs. 0.58; *F*(1, 37) = 11.44, $BF_{10}$ = 17.50, $\eta ^2_G$ = 0.21]. The interaction effect was not substantial [*F*(1, 37) = 3.19, $BF_{10}$ = 0.97, $\eta ^2_G$ = 0.01].

## Discussion

This study examined how task load and interruption frequency affected the allocation of attentional resources measured via novel numerosity measure of eye movements, GTE and SGE, in a multitasking environment. Previous experiments used PDT as a measure of attentional resources^17,18,29^, which may not necessarily capture dynamic transitions between AOIs in such a multitasking operational environment. For example, a simple analysis of PDT cannot diagnose whether operators’ eye movement patterns became more random or predictable in response to an elevated level of task load. As such, we applied gaze transition entropy analyses to Sato et al.’s^18^ data.

Corroborating with the original analysis of Sato et al.^17^, the results of SGE showed participants’ fixations during each experimental session were more concentrated and predictable under the Difficult than the Easy condition. This further lends support to the conclusion of Sato et al.^18^ that operators attended behaviors of the automation more systematically while mostly focusing on the tracking task with greater difficulty. Moreover, GTE analysis showed their eye movements transitioned across AOIs more predictably in the Difficult condition where the tracking task required more frequent manual correction than in the Easy condition. These data imply that operators change their scanning strategies by restricting both spatial dispersion and dynamic shift of attention when their task load increases.

More interestingly, the analyses revealed that participants’ eye movements became more random in the High than Low communication frequency condition. More specifically, SGE was greater when participants were asked to respond to 16 interruptions than 4 interruptions while performing the other two concurrent tasks, suggesting more spatial dispersion of fixations in the High communication frequency condition. There are at least two possible accounts. First, participants showed greater entropy because they made fixations more frequently to the display for the communication task in response to the interruptions in the High than Low communication frequency condition. That is, SGE might have increased simply because participants reactively moved their eyes to perform the required task when needed. Second, participants’ SGE increased because participants proactively scanned the display for the communication task in anticipation of the possible onset of auditory stimuli for the communication task. It is unlikely that only 12 communication trials of 30 seconds each will substantially affect a more global measure of spatial dispersion of fixations over an experimental trial of 20 minutes. Instead, it is likely that participants adopted a strategy to broadly scan the three tasks with some emphasis to the display for the communication task when they experienced the communication task interrupted them frequently. Partly supporting this view, GTE was also greater in the high than low interruption frequency condition. This reflects that participants’ eyes transitioned between AOIs in a less predictable manner. If the first account above is correct, then we should observe GTE lower in the high than low interruption frequency condition, because the shift of fixations to the communication task in response to the auditory stimuli is highly predictable and less frequent. Instead, the current data accords with a view that participants checked the communication task more frequently when more interruptions occurred.

Why did the communication frequency manipulation increase gaze entropy while the task load manipulation decreased it? A possible account is that the communication task required task switching while the tracking task did not. Because the communication task arose in a more discrete manner, their attention is likely to temporarily shift to the communication task when the auditory stimuli were presented while suspending an ongoing task (the tracking or system monitoring task). According to the Strategic Task Overload Model (STOM)^30^, the probability of a task being switched to is a function of four parameters. *Priority* is an overall importance of the task and is analogous to the value parameter in SEEV. *Difficulty* refers to the mental workload that the task imposes and is analogous to the effort parameter in SEEV. *Salience* is a relative perceptual distinctiveness that attracts one’s attention in a bottom-up manner and the same in SEEV. Finally, *interest* is the attractiveness of a task-irrelevant to the formal task requirement and is absent in SEEV. The current communication task received an equal priority compared to the other two tasks and the difficulty level is relatively higher because the task requires both visual and auditory modalities and careful interpretation of noisy auditory messages. The task is more salient because the auditory stimuli arrive unpredictably and occur less frequently than the other tracking and system monitoring tasks. It is likely that the communication task attracts their attention more heavily from the other two tasks, potentially changing overall task priority or interest parameters giving a lasting effect on overall visual scanning strategies.

Results highlight that gaze entropy analysis, using SGE and GTE, provides a new window into attentional resource allocation in a multitasking workspace. SGE and GTE provide summary statistics of spatial dispersion and predictability of attentional shifts across AOIs, providing a novel and useful measure of resource allocation. These calculation algorithms are grounded in Information Theory and are relatively easily implemented. In practice, automation designers may incorporate gaze entropy analysis to reveal the underlying attentional strategies of the users and infer their cognitive state based on eye movement patterns.

## Methods

Sato et al.^18^ recruited forty undergraduate students (29 females and 11 males; *M* = 20.03 years, *SD* = 2.72 years) from the community of Old Dominion University, Virginia. Participants had normal or corrected-to-normal vision and normal color perception. Participants received course credit for participation.

We applied GTE and SGE analyses to the data of Sato and his colleagues^18^. In their task, participants were asked to perform three concurrent tasks in the Multi-Attribute Task Battery (MATB-II^31^)—the compensatory tracking task, system monitoring task, and communication task. In the tracking task, participants used a joystick to keep the moving circular target within the dotted square. The frequency of force function of the target’s random movement was manipulated to generate the high load condition (0.12 Hz) and the low load condition (0.06 Hz) similar to Karpinsky et al.’s^29^ study. In the system monitoring task, participants were asked to monitor four vertical gauges. Within each vertical gauge, the yellow pointer fluctuated between the center of the vertical gauge. When the pointer hits either the top or bottom extremity, representing system malfunction, participants were instructed to correct the yellow pointer by pressing one of the four keys (F1–F4) relative to the four vertical gauges. The system monitoring task was supported by an automated signaling system with 70% reliability. The signaling system issued a green light above the four vertical gauges when all the engines are in normal state and a red light when abnormal. When the signaling system issued a warning, participants were instructed to respond by turning off the red light (press F6) and turning on the green light (press F5). The reliability of the signaling system was controlled based on the correct response (Hit) and false alarm (FA) events. In a Hit event, the signaling system correctly detects system malfunction. In an FA event, the signaling system issues a warning even though there is no system malfunction. All blocks comprised of 28 Hit events and 12 FA events that occurred at random time intervals. In the communication task, participants used a mouse to change the frequency of a relevant radio according to an auditory message.

Sato et al.^18^ used a 2 × 2 design with Communication Frequency (High vs. Low) as a between-subject factor and Difficulty (Easy vs. Difficult) as a within-subject factor. Each participant completed two 20-minute experimental trials performing the tracking and system monitoring task while processing either 4 or 16 auditory messages in the low and high communication frequency conditions, respectively.

**Fig. 4:**
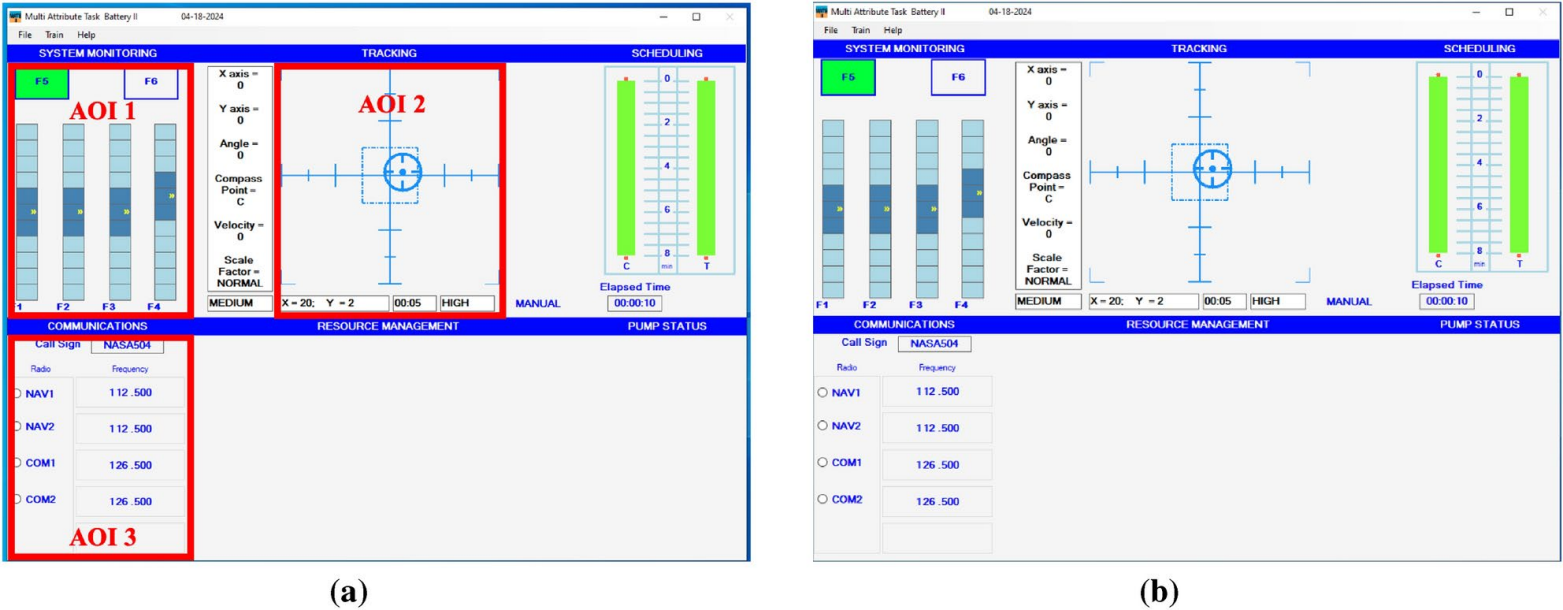
Sample display of the MATB-II environment with three AOIs used for the analysis (**a**) and without AOIs (**b**).

Eye movement data were reanalyzed to calculate GTE and SGE using *Eye Tracking Metrics Calculator* in Python^32^. GTE indicates the extent to which gaze transition is uncertain with a higher value indicating an unpredictable fixation pattern, while SGE indicates the extent to which gaze transitions are spatially distributed with a higher value indicating equal distribution of fixation across AOIs. Following Sato et al.^18^, the present study defined AOIs relative to the size of each task. AOIs are defined in Fig. 4. We employed default Bayesian analysis with Bayes factor as a measure of evidence^33^, denoted as $BF_{10}$. Bayes factors were interpreted according to Jeffreys^34^.

All methods were carried out in accordance with relevant guidelines and regulations. All experimental protocols were approved by the College of Sciences Institutional Review Board at Old Dominion University, and informed consent was obtained from all participants.

## Data Availability

The datasets used and/or analysed during the current study available from the corresponding author on reasonable request.
